# Psychological Experience of Humanistic Care Among Medical Staff in Stroke Wards: A Qualitative Research Study Conducted in China

**DOI:** 10.3389/fpsyt.2022.791993

**Published:** 2022-03-25

**Authors:** Min Li, Wen-jing Zhu, Qing Luo, Huang Chen, Yan Duan, Hong-zhen Xie

**Affiliations:** ^1^Department of Nursing, Chongqing University Central Hospital, Chongqing, China; ^2^School of Nursing, Guangdong Pharmaceutical University, Guangzhou, China; ^3^Department of Neurosurgery, General Hospital of Southern Theatre Command, Guangzhou, China; ^4^Department of Endocrinology, General Hospital of Southern Theatre Command, Guangzhou, China; ^5^Department of Emergency, General Hospital of Southern Theatre Command, Guangzhou, China; ^6^Department of Health Medicine, General Hospital of Southern Theatre Command, Guangzhou, China

**Keywords:** stroke, stroke ward, humanistic care, physiological experience, qualitative research, nursing humanistic care

## Abstract

**Background:**

As a special patient group, stroke patients have a significant attachment to humanistic care. However, multiple problems remain in clinical practice. Medical staff in stroke wards are the primary providers of humanistic care. Finding out the opinions of the staff that provide these medical services is vitally important for stroke patients that need access to curative and humanistic care.

**Objective:**

The aim of the study is to explore the psychological experiences of doctors, nurses, and physiotherapists during the implementation of humanistic care in stroke wards.

**Method:**

This is a qualitative phenomenological study. Medical staff (i.e., doctors, nurses, and physiotherapists) were selected from stroke wards in general hospitals (minimum level two) from 13 cities within six provinces in China. A purposive sampling method was used until saturation (*n* = 18). Face-to-face or video call semi-structured interviews were conducted by using a phenomenological research method. The average interview length was 60 min (range 30–90 min). The Colaizzi seven-step method was used for analysis.

**Results:**

Four themes and 12 sub-themes were extracted from the qualitative interviews of the medicine, nursing and technology staff, as follows. ➀ The ward staff reported that the behaviors of the stroke patients gradually improved when they assisted with stroke treatment idea changes, when they paid attention to solving the patients' existing problems, and when they took the initiative to create a caring atmosphere; ➁ when humanistic care in the stroke wards was carried out with consciousness and ability improvement (including proactive caring behaviors in which vocational value was not strong and in which the whole-person rehabilitation was given attention, not just implementation), the patients' behaviors improved; ➂ the stroke wards themselves were improved (the gap between the current management and the needs of medical institutions and the gap between the rehabilitation conditions and the patients' needs were addressed); and ➃ the urgent needs of the staff in the implementation of humanistic care in stroke wards were considered (the addition of full-time posts, the effective training of humanistic care, and the construction of a more harmonious doctor–patient relationship).

**Conclusion:**

In implementing humanistic care in stroke wards, the consciousness and ability of the medical staff need to be improved. In addition, the practical problems and contradictions affecting the development of humanistic care must be addressed. To improve the level of humanistic care in stroke wards, attention should be paid to the overall improvement of the personal qualities of the medical staff and the integration of a humanistic management mode.

## Background

Stroke is a threat to the lives and health of the general public, with the significant characteristics of sudden onset, wide influencing factors, multiple complications and high recurrence rate (1). According to the data, there are about 2.4 million first-episode stroke patients and 1.1 million deaths in China every year, and only about 11 million post-stroke survivors at any time, ranking first in the list of causes of death in China and in the top three in the global causes of death (1–3). About 75% of the survivors of stroke have functional and psychological disorders (4). Neurologic deficits and self-role changes are clearly noticeable for cerebral apoplexy patients with psychological pressure surge, including (5) shame after the illness, and anxiety, depression and other negative emotions. In addition, a variety of mood disorders and extreme psychological illnesses appearing after stroke can result in self-injury and suicide (6) or a series of social problems. This affects the patients' quality of life (7–11) and increases the economic pressure and care burden of family and society (12, 13).

The origin of the western word “humanity” is the Latin word “humans,” indicating everything that is human-centered; in China, it originated from the *BOOK of Changes*, aiming to cure diseases and save people as its most beneficial principle (14). In professional relationships, based on their own quality, medical staff will spontaneously transfer the caring concept of the “whole person” to patients in vulnerable states, turning the professional relationship into one that is more like family, demonstrating behaviors that will make patients feel loved and cared for (15). It emphasizes the reverence for life and the need for medical staff to improve their humanistic qualities based on sufficient professional knowledge. This spirit should be integrated into medical practice to give timely and effective treatment to patients to remove or alleviate their pain and to give them enough respect and compassion to humanely meet their reasonable needs.

Encouraged by the International Circulation Care Association, 42 Guidelines for Humanistic Care for nurses were issued in 2003 (16). In the same year, Watson created the ANCM model from the perspective of patients to improve nurses' caring ability by providing nurses with programs that included care assessment, planning, implementation and the establishment and maintenance of continuous care (17). Chinese humanistic expert Liu Yilan's team (18–22) drew on Watson's 10 caring elements to carry out humanistic nursing practice research for the purpose of caring management. In the form of theoretical guidance, caring ward pilots, thematic research, international cooperation and industrial exchange, the humanistic care model for inpatients was constructed and the construction of relevant evaluation indicators was improved, providing direction guidance for nursing managers' care management.

Studies have shown that caring nursing can promote the acquisition of a helpful caring experience for stroke patients, while non-caring nursing promotes the opposite (23). Humanistic care not only improves the physical and mental health status of stroke patients but also affects the recovery outcomes (8). It is an important measure to improve the quality of nursing service in stroke wards. Rejn and Berg (24), based on dignity care, fully highlighted the patient-centered nursing thought, encouraged patients with acute stroke who could not defend their own interests to express their personal wishes and assisted them to achieve this through the nursing staff and close relatives. SALK Hospital Clinic in Salzburg, Austria (25) cares for patients with post-stroke dysfunction as if they were normal and treats each patient in a child-like way. At the same time, nurse-led care has been gradually developed in China, aiming to reduce the disability problems of stroke patients and help them return to society to the maximum extent. Deng (26) used action research and the Delphi method to construct a case management model for stroke patients with risk factors, complications and rehabilitation management as the core content to promote the neurological rehabilitation of stroke patients. Chen (27) established the rehabilitation nursing path for stroke patients with the clinical nursing path and improved the early rehabilitation consciousness and behavioral ability of nurses in the stroke ward. However, there is currently no uniform standard to evaluate medical humanistic care. Most of the care processes depend on the conscious awareness of managers and the personal qualities of the implementors (2, 4). The practical status is not optimistic.

To reduce the pressure of public health, to help the patients establish good rehabilitation confidence and disease response capacity, to promote patients' maximum return to society, and to improve their survival and quality of life after stroke, humanistic care should be implemented across China. This study is adapted from the programs in *Health of China's 2030* (28) and *The Development of Chinese Nursing Career Planning Outline (2016–2020)* (29). Based on the requirements of the national policy that medical work should be integrated with humanistic care and that medical service should enhance the construction of a humanistic care system, this study is conducted to understand the perception of stroke ward staff on the implementation of humanistic care at its present stage and to construct humanistic care guidance for stroke patients in the future.

This study will fully consider the experiences of both the care implementors and the care recipients. As the principal part of humanistic care practice in stroke wards, the feelings of the medical staff can affect and even determine their own caring behaviors. To thoroughly investigate the details and problems associated with the implementation of humanistic care in stroke wards, this study explores the psychological experiences of doctors, nurses and therapists through semi-structured interviews, with the goal being to improve the overall level of care.

## Methods

### Design

This was a qualitative phenomenological study. A phenomenological method and thematic analysis were used within qualitative face-to-face or video call semi-structured interviews, both of which are part of the practice guideline structure for stroke care. Before starting, the research group defined the practical meaning of humanistic care for stroke survivors. The expertise group consisted of a nursing methodology professor, a director of the hospital nursing department, a director of the neurology department and a chief nurse. This expertise group conducted a feasibility study and quality control analysis regarding the processes of topic selection, study design and potential problem finding in this draft study and put forward suggestions for revisions.

Two interviewees were involved in each interview. The main interviewer was the systematic literature evaluation specialist authorized by the Joanna Briggs Institute, who had nearly 10 years of clinical work experience and was competent in the use of communication and interview skills. The interviewer maintained a neutral attitude throughout the interview, without inducing or hinting to obtain statements from the interviewees. The analytical process was suspended, and things were evaluated with an open mind, so the interviewer was not influenced by their existing knowledge and experience (30). SRQR reporting specifications were used to present the research results, and the quality evaluation method of qualitative research was used to review the level of rigor applied.

### Participants

The study was conducted from September–October 2019. The participants were from stroke wards in general hospitals (graded level two or above) from 13 cities within six provinces engaged in an advanced study in the department of neurology and neurological rehabilitation at a comprehensive hospital in Guangzhou, China. The inclusion criteria were as follows: (a) certified and valid registered doctors, nurses and rehabilitation technicians, with (b) formal labor relations with the employing unit, having (c) more than 3 years' experience in a stroke ward and (d) providing voluntary consent. People that had left their position for over a year and non-clinical positions (e.g., management positions and the logistics department) were excluded. Recruitment ceased when data saturation was reached, meaning that no new information relating to the participants' perceptions of humanistic care was noted in the transcripts.

### Data Collection

Based on the previous research, an outline was made of the interviews following the group discussion, a consultation with the experts and a pre-interview. The semi-structured interviews included four main questions (Table 1).

**Table 1:** Interview questions.

**No**.	**Question**
1	What's your opinion on the current implementation of the humanistic care in stroke ward?
2	How do you feel about the humanistic care implemented by the stroke department?
3	What are the obstacles of the implementation of the humanistic care in the stroke ward? How do you manage those difficulties?
4	What strategies can help to improve the level of humanistic care in stoke ward?

All the participants were briefed about the study and informed of their rights and obligations in the study. The first author made appointments with the participants after they acquired written consent for the interview from the participants. To encourage the participants to describe specific experiences of humanistic care in stroke wards, follow-up questions were asked later by the researcher. The average interview length was 60 min (range 30–90 min), and an audio recording was made of each interview.

### Ethical Considerations

This study was approved by the Ethics Committee of the General Hospital of the Southern Theatre Command of the Chinese People's Liberation Army. A full explanation of the research purpose and method, rights and obligations, use of recording equipment and other related matters was given to the interviewees. All participants provided a written and signed letter of consent to be interviewed.

### Data Analysis

Colaizzi's data analysis method was used for clustering the themes from the known phenomenon (31). Every audio recording was transcribed verbatim and reviewed as soon as it was finished by the first author (ML). All the transcripts were read multiple times by the first authors to reach a common understanding of the perceptions of the participants and gain a deeper understanding of the content. Then, the first authors continued the data analysis process by labeling the data extracts from all the interviews with a coding system.

## Results

### General Participant Information

Eighteen participants were recruited, including five doctors, eight nurses, and five rehabilitation technicians. Their average age was 36.66 ± 5.57 years. Their average work experience time was 11.83 ± 6.82 years. One of the participants was a follower of Islam, with the others having no religious beliefs. Table 2 shows the detailed characteristics of the participants.

**Table 2:** Characteristic of participants.

**No**.	**Gender^a^**	**Age**	**Education level**	**Work place (City, Province)^b^**	**Length of care**	**Professional title**	**Unit level^c^**
a1	M	36	Master	Huizhou, GD	8	Advanced	3rd
a2	M	35	Bachelor	Heshan, GD	12	Advanced	2nd
a3	M	31	Master	Guangzhou, GD	4	Junior	3rd
a4	M	33	Bachelor	Zhanjiang, GD	9	Intermediate	3rd
a5	F	32	Doctor	Guangzhou, GD	5	Intermediate	3rd
b1	F	38	College	Panzhihua, SC	19	Intermediate	3rd
b2	F	39	Bachelor	Zengcheng, GD	15	Intermediate	3rd
b3	F	38	College	Maoming, GD	17	Intermediate	2nd
b4	F	46	College	Guangzhou, GD	24	Intermediate	3rd
b5	F	30	Bachelor	Bengbu, AH	10	Junior	3rd
b6	F	28	Bachelor	Suzhou, AH	7	Junior	3rd
b7	F	29	Bachelor	Nanning, GX	7	Junior	3rd
b8	F	33	Doctor	Guangzhou, GD	6	Advanced	3rd
c1	F	35	Master	Guangzhou, GD	16	Intermediate	3rd
c2	M	32	Bachelor	Shenzhen, GD	13	Intermediate	3rd
c3	M	33	Bachelor	Yinchuan, NX	8	Intermediate	3rd
c4	F	48	Bachelor	Guangzhou, GD	28	Advanced	3rd
c5	M	28	Bachelor	Ganzhou, JX	5	Junior	3rd

a *M, Male; F, Female*.

b *GD, Guangdong Province; SC, Sichuan Province; AH, Anhui Province; GX, Guangxi Province*.

c *3rd, 3rd level hospital; 2nd, 2nd level hospital*.

### Themes Revealed

Four main themes were extracted from the manuscript: “Gradually improving the perception of humanistic caring,” “Required improvement of consciousness and ability of humanistic care implementors in stroke wards,” “Main problems and principal contradictions in implementing humanistic care in stroke wards,” and “Critical needs of staff in stroke wards.” Table 3 displays both the prime motivators and obstructions to implementing humanistic care in stroke wards.

**Table 3:** Summary of themes, coding words, and meaningful units.

**Main themes**	**Sub-themes**	**Coding words**	**Meaningful units and professional**
Gradually improving the perception of humanistic caring in stroke ward	Changes in concept of treatment	Stroke ward	a1, Doctor
		Green channel of stroke	a5, Doctor
		High quality of nursing care	b1, Nurse
		Respect for human rights	b3, Nurse
		Spirituality concern	b4, Nurse
	Solve patients' problem	Dysphagia treatment	b1, Nurse
		Pay for patients' care	b8, Nurse
		Deal with severe constipation and urinary retention	c2, Physiotherapist
			c4, Physiotherapist
	Initiatively create caring atmosphere	Comfort in holiday	a1, Doctor
		Build communication platform	b7, Nurse
		Interaction of medical workers and patients	c2, Physiotherapist
Required improvement of consciousness and ability of humanistic care implementors in stroke ward	Weak professional value	Distraction of self-emotion on work	b8, Nurse
		Disengagement of work	c4, Physiotherapist
	Insufficient voluntary caring behavior in stroke ward	Neglect of coma patients' care	a3, Doctor
		Perfunctory education	b5, Nurse
		Indifferent care attitude	c5, Physiotherapist
	Inadequate attention and capacity on whole-process rehabilitation	Lacking early rehabilitation	b1, Nurse
		Indifferent to rehabilitation	b3, Nurse
		Lack of comprehensive skills	a2, Doctor
			b2, Nurse
			b7, Nurse
Main problems and contradiction in implementing humanistic care in stroke wards	Contradiction between reality and demand in management of medical institution	Unbalance allocation of human resource	b1, Nurse b6, Nurse
		Unreasonable work flows	b3, Nurse
		Conflict between principle and flexibility in management	b5, Nurse
	Disparity between rehabilitation conditions and patients' need	Tight amount of beds	a2, Doctor
		Incomplete rehabilitation medical construction	c2 Physiotherapist
Critical need of staff in stroke ward	Additional position setup	Necessary position of psychological consultation	c1 Physiotherapist
		Position of physiotherapists	b6, Nurse
	Effective practical training of humanistic care	Cases and experiences of humanistic care	a4, Doctor
		Training does not fit the reality	b1, Nurse
		Ambiguous content of care	b5, Nurse
	Build a more harmonious relationship between patients and doctors	Rigid cognitive	a1, Doctor
			b5, Nurse
		Poor compliance	a2, Doctor
		Lack of trust	c3, Physiotherapist

### Theme 1: Gradually Improving the Perception of Humanistic Caring

#### Changes in Concept of Treatment

Due to the widespread nature of the stroke prevalence areas, inconsistent service levels and non-standard stroke prevention rescue systems, the National Health Commission has established a stroke specialist management system and formed an integrated process of pre-hospital treatment, family recuperation and social rehabilitation by integrating multi-disciplinary advantages. This system has changed the traditional pattern of stroke care. In the study, eight participants described how the treatment mode has transformed into multi-disciplinary cooperation, improving the conscious awareness of humanistic care among doctors, nurses and technicians:

“*The construction of stroke units has become more mature in our country, which makes a big difference in the care of patients.” (a1, a5)*

“*Nursing patterns [have] changed a lot these days, which also put[s] new demand on us…we need to be aware of all aspects of [the] patients.” (b3)*

“*Some relatives of seriously ill patients put the national flag like a bedspread on the bed and play some scriptures that we could not understand. In the past, we would certainly try to stop them, but now we will not intervene too much.” (b4)*

#### Solving Patients' Problems

Ten participants agreed that fully addressing the specific problems of dysfunction and hospitalization is an important way for them to fully experience the core components of humanistic care:

“*I have treated a patient with dysphagia very well, and he said I had been his benefactor all his life.” (b1)*

“*The patient couldn't sleep because the light was too bright, so the nurse bought the patient an eye mask at her own expense.” (b8)*

“*A post-burn stroke patient was transferred from the primary hospital. After our efforts, his 2-month-long urine and gastric tubes had been removed. He sent us a pennant.” (c4)*

#### Initially Creating a Caring Atmosphere

Creating a harmonious and caring atmosphere is an essential condition for the humanistic care of stroke patients, enabling patients to feel comfortable in the medical environment and enhancing their sense of social participation. It is mentioned by all participating professionals, and they all took it seriously:

“*On some major holidays or their birthdays, we will give gifts or* throw *parties for patients who cannot be discharged from the hospital, so that they can feel the warmth of home in an unfamiliar environment.” (a1)*

“*[If] there are patients in our department with similar hobbies, then we will introduce them to each other.” (b7)*

“*Our director used his rest time to visit the patients and do crafts together…” (c2)*

### Theme 2: Required Improvement of Conscious Awareness and Ability of Humanistic Care Implementors in Stroke Wards

#### Weak Professional Value

Consciousness is the subjective impression of objective things, and practice is the internal power of consciousness development. They influence each other and demonstrate reciprocal causation. Doctors are not only the behavioral subjects of medical services but also the holders of different social roles. Multiple pressures will directly affect their loyalty, sense of responsibility, dedication and sense of achievement, all of which will be transferred to the patients through medical services. Among the participants, seven of them said that their professional honor makes them feel more comfortable, six of them said they only work to support their families, and five complained about their career choice.

#### Insufficient Voluntary Caring Behavior in Stroke Ward

Too much attention is paid to the treatment of the condition instead of the treatment of the person. Six of the participants said that humanistic care was highlighted for many reasons (i.e., insufficient conscious care behavior of the staff, caring carried out by the escorts and coping with education):

“*We couldn't even perform [humanistic care] on lucid [patients], let alone those* in a coma. *What's the point of the care?” (a3)*

“*At present, patients can only be briefly introduced to the ward environment during admission evaluation, there is nothing else.” (b5)*

#### Inadequate Attention and Capacity for Whole-Process Rehabilitation

Self-realization places the highest demand on individuals in Maslow's hierarchy of needs, and rehabilitation is an important way for patients to return to society. The lack of a rehabilitation concept and the required comprehensive skills will increase patients' resistance to forming social ties. Nine respondents said that they felt guilty due to insufficient awareness, implementation and follow-up guidance. In the interview, multiple interviewees also reflected on the impact of inadequate competence, including professional competence and humanistic practice on the rehabilitation process of stroke patients.

“*Because we did not do well in early recovery, a sober patient [contracted] and shrunk like a ball (because of the complication). Every time I passed his ward, I could not bear to see him (*tears in eyes*).” (b1)*

“*Rehabilitation is so important. But we just overlook it. We don't know whether it do*es *good to [the] patient and whether there is a difference in the treatment.” (b3)*

### Theme 3: Main Problems and Principal Contradictions in Implementing Humanistic Care in Stroke Wards

#### The Contradiction Between Reality and Demand in the Management of Medical Institutions

In this study, several participants said that humanistic care attributes were not taken into account in staff selection, existing work processes and management systems, resulting in patients not benefitting. Instead, multiple complaints were lodged, and the staff themselves felt weary and helpless:

“*The average number of hospitalized patients is more than 170, with only* ~*20 nurses, and the corridor is full of camp beds…” (b1)*

“*We have to go around and make an inspection every hour. Points will be deducted if the rounds are too short. Patients are disgusted that they cannot get to sleep easily and then are woken up by our rounds [helpless sigh].” (b5)*

“*Some people who are not paraplegic can sit up and move moderately with the help of others, but we really cannot spare the time and energy to help them [helpless].” (b6)*

#### Disparity Between Rehabilitation Conditions and Patients' Needs

The social security mechanism and the medical rehabilitation system are gradually improving in China. However, due to past restrictions, an uneven distribution of high-quality medical resources, insufficient total amount, etc., stroke rehabilitation conditions do not meet the requirements of patients.

“*Despite the expansion of hospital areas or the construction of new hospitals every year, the number of rehabilitation beds is still not enough, and [they are] always full…The patient can't make an appointment for [a] bed, so the rehabilitation treatment is suspended.” (a1)*

“*The framework for rehabilitation is not yet fully established…It is difficult for therapists to carry out continuous treatment independently of hospitals.” (c2)*

“*A lot of patients line up at 7 am to get a place for physiotherapy…The corridor was jammed and there was a lot of noise.” (b3)*

### Theme 4: Critical Needs of Staff in Stroke Wards

#### Additional Position Setup

Compared with other departments, the workload of the staff in stroke wards is larger, as patients suffer from serious illness and have prominent psychological problems that require significant rehabilitation needs. Most medical staff hope to change the status of the stroke wards through post rearrangement or seamless connection of various departments. The two most urgently needed positions are rehabilitation therapists and psychological counselors.

#### Effective and Practical Training for Staff in Humanistic Care

The characteristics most referred to by the participants are as follows: reservations about lack of self-knowledge, confusion over the solid implementation of humanistic care and a desire for caring guidance. Behavioral guidance and caring paradigms are what they really need.

“*Usually there is no special class or training for communication skills, humanistic measures and so on…We won't know what to do.” (b1)*

“*I was the leader of the humanities group in the ward, but I was confused on what to do with humanistic care…” (b5)*

#### Build a More Harmonious Relationship Between Patients and Doctors

A good doctor–patient relationship is not only the premise for mutual trust and close cooperation between doctors and patients but is also the basis of humanistic care. The stereotypical image and false impression of stroke by patients or their families lead to blocked communication between doctors and patients; in turn, this affects the harmony between doctors and patients, bringing greater challenges to the implementation of humanistic care. This study's participants were frustrated with the behavior of the patients or their family members:

“*Some patients or family members are very anxious, asked for transfer when the effect is not so significant, admitted to the hospital 1–2 days, looking at the effect is not so obvious immediately, completely not explained…” (a1)*

“*At the beginning, he took the medicine on time, but as soon as he saw the improvement, he stopped. He relapsed again and again, and his condition became worse. It was because of our poor skills that he failed to meet his expectation of recovery.” (a2)*

“*Many people do not trust the primary hospitals and even think it is a waste of money to go there. Some of them will guard against medical staffs, so we hardly dare give our hearts to them in the process of care.” (c3)*

## Discussion

### Improvement of the Treatment Process Is the Key to Promoting the Humanistic Experience of Patients

Time is the brain. Early endovascular therapy saves lives and reduces disability, which can then maximize the patients' opportunity to return to society. Therefore, the establishment of the humanistic stroke rescue process is the basis of humanistic care. The Canadian Stroke Best Practice Recommendations for Acute Stroke Management (32) recommend that emergency centers should speed up the thrombolytic process through multidisciplinary collaboration, joint networking centers, optimisation of pre-hospital care procedures, and opening of green channels. The establishment of a green channel for emergency stroke care is the first step in process improvement, from strengthening the stroke identification ability to reducing the disease process. By moving the treatment site forward, the time from arrival to treatment initiation (door-to-needle) can be greatly shortened and the treatment efficiency can be improved.

This study concluded that the idea of a single stroke department treatment center had a subtle effect on stroke ward workers, and the concept of the service was also changed accordingly. In addition to actively creating a caring atmosphere and taking care of the patients' personalized needs, the most influential factor was the establishment of a rapid response medical channel. Stroke centers in China are run *via* multidisciplinary medical modes, led by disease category (33). They effectively integrate the resources and normalize stroke treatment through the whole process. In addition, they have standardized the management of stroke diagnosis. The treatment process should be reorganized according to the needs of specific diseases, and a humanistic treatment process should be established to provide patients with faster, safer and fairer services so that the medical personnel can participate effectively based on providing good treatment. Furthermore, the role of the nurse should be fully highlighted. As the most capable person to perform humanistic care, the nurse can not only give priority to the care of emergency patients but can also act as the process coordinator, guiding and integrating medical resources (34). Care should be integrated into the details of the process; this would improve the humanistic care of patients by effectively attending to their needs.

### Improving the Ability of Personnel Is the Foundation of Humanistic Care

Comprehensively improving the humanistic quality of the medical staff is the basis of implementing humanistic care. In several continuing education training programmes, 60% of the content is aimed at improving medical behavior rather than focusing on the patients (35). Most interviewees stated that “The failure to pay comprehensive attention to patients in clinical work is mostly related to the lack of caring awareness and ability. Worse still, there are not enough effective knowledge and skill training programmes in hospitals. As a result, their knowledge structure does not meet the needs of patients' care needs.” Related studies (36, 37) have shown that it is difficult for some medical staff to truly understand patients, with poor overall care consciousness, insufficient communication skills and low humanistic practice ability. The proportion of medical disputes caused by humanistic factors, such as service attitude, service language, and medical ethics, is as high as 80% (an important driving force in affecting the patients' level of satisfaction with the care received) (38).

Researchers believe that the education and management of care can be important factors in improving the quality of care from workers (39). Therefore, it is vital that medicine, humanistic care and personal qualities be integrated to provide continued education. To enhance the professional values of the medical staff, training programmes should include professional basic knowledge and information on humanistic ability. A further influence is motivating them to consciously have a people-oriented attitude in their work (40).

It was reported that, when using the same recognized rate of stroke patients among doctors and nurses, there is a difference in care as nurses take more prompt action. Strengthening the professional basic training of nurses is of great importance. The curriculum content should be designed according to varying levels and targeted toward collaborative education. This could further improve the prognosis for stroke patients. In terms of training methods, repetitive active knowledge uptake (41) and diversified teaching situations (42, 43) are more conducive to improving the comprehensive care ability of doctors, nurses, and therapists in stroke wards.

### Optimizing Stroke Ward Management Systems Is a Guarantee of a Humanistic Care Service

A people-oriented approach to management is the key to improving care in stroke wards. The research results show that some medical institutions with insufficiently humanized management can lead to unreasonable working processes and systems, poor stability of the medical team, insufficient cohesion, and difficulty in demonstrating professional value. With the prevailing national conditions of unbalanced doctor–patient ratios and imperfect rehabilitation, limited care time is given to patients. Stroke patients have obvious physical, functional, and emotional disorders. Doctors and nurses in stroke wards with inadequate knowledge of professional rehabilitation treatment or psychological consultation find it difficult to meet the diverse needs of stroke patients, posing even greater challenges to the development of humanistic care.

A collaborative method, put forward by the American Association of Critical Care Nurses, has been widely used in nursing management (44–46). From a management perspective, it advocates reasonably equipping human resources by matching the needs of the patients with the ability of the nurses and clinical systems, forming a corresponding nursing service process and management system based on the requirements of patients.

Therefore, it essential to optimize the combination of medical workers and set up the necessary specialist positions (e.g., rehabilitation therapists, psychological counselors, case managers, and specialist nurses) according to the stroke specialty characteristics (47). This makes a positive difference and improves their professional value, positively impacting their job satisfaction and their caring effectiveness. Meanwhile, managers and employees should jointly shape the organizational culture and set a shared vision for motivation. Managers should also make full use of “leading by example” to provide a visible model of behavior so that employees can be led toward a better professional ethos; in turn, this will consequently build a better type of hospital and create a more harmonious medical environment (48).

### Limitations and Deficiencies of This Study

This study is a phenomenological study, and the purpose sampling method was adopted in line with the research program. It is well-known that the disadvantage of this sampling method is that the results are greatly affected by the researchers' tendencies. Once there is subjective judgment bias, sampling bias is easily caused. It is not possible to completely extrapolate this bias from the survey. In this study, the researchers did conduct strict quality controls to minimize any bias. To ensure that the respondents were representative of the uniform distribution of the sample population with different characteristics, basic data (i.e., age and educational background) were fully considered in the selection criteria. Before the interview, the interviewer established a good relationship with the interviewee in which the interviewer attempted to fully understand each medical employee who was an interviewee, conducted an in-depth investigation and necessary analysis on the basic information and language expression of the interviewee and extensively collected reflections and opinions from all aspects. Although it was strictly controlled, from the perspective of the selection of the research participants, the individuals from tertiary hospitals still accounted for the overwhelming majority. In the future, additional data from hospitals and community units below the tertiary level can be obtained for more comprehensive information. However, this issue does not affect the reliability of this study's results. The interviewer took a neutral attitude throughout the interviews, without inducing or suggestive statements, and encouraged the interviewees to fully express themselves by clarifying inaccurate or unclear information, by recording the expressions and non-verbal behaviors of the participants, and by stopping the interview when information saturation was reached. The study was rigorous from the point of view of discontinued sample inclusion. At the end of the interview, the relevant materials were summarized and sorted out, and the analysis process was conducted without personal preference. The researchers looked at things with an open horizon without interference from existing knowledge and experience and mined the effective information with the help of computers. A repeated study of the recordings, careful transcription and analysis. After the interview was transcribed, the transcription was compared with the original recording and then sent to another party for confirmation. Through the above steps, the inevitable bias problem in the sample selection was dealt with to the maximum extent possible.

## Conclusion

Through in-depth interviews with 18 respondents in 13 cities in China, this study found that the implementation of humanistic care in stroke wards was not an optimistic experience from the perspective of medical staff.

Their main psychological experience includes the fact that currently, the caring behavior toward patients in the stroke ward is reflected in medical practice but that the caring consciousness and the ability of the providers to provide humanistic care cannot fully meet the patients' needs; this needs to be improved upon. This is specifically reflected in the fact that the personal professional value sense is not strong. Therefore, the implementation of active care behavior is not enough, and the whole-person process of rehabilitation is lacking attention. This study's participants also felt that there were multiple practical difficulties in the implementation of humanistic care in stroke wards (i.e., the large gap between the current management and the rehabilitation conditions of medical institutions and the patients' needs). In the future, they expect to add physical therapists, psychological consultants, and other full-time posts in the stroke ward; these new medical staff members are expected to receive effective humanistic care training and assist in building a more harmonious doctor-patient relationship. According to this study's results, although the caring consciousness of the staff in the stroke ward has been improved with the change in the stroke treatment process, multiple deficiencies remain in the overall care concept. There are several influencing factors for this; the most prominent one is that the patients' needs cannot be met. The medical psychological feelings obtained in this study once again verified the necessity of constructing humanistic nursing practice guidelines for cerebral apoplexy. In addition, the implementation of care guidance is conducive to the improvement of the service quality in the stroke ward. The respondents believe that the common concern of the patients is to return to society; therefore, how to help these patients recover quickly should be the fundamental purpose of humanistic care. Another main purpose is to reflect specialty characteristics and professional standards in humanistic nursing practice guidance. This finding provides a possible direction for the construction of the humanistic nursing practice program. The main purposes of this program should be to enhance the awareness of care and build care management as a supplement. Based on professional considerations and combined with the needs of the patients and the characteristics of the disease, the goal is to return these patients to society.

Through their medical treatment behavior, medical staff should be keen to capture the psychological responses and the needs of their patients to solve practical problems for them based on the characteristics of their condition. Medical staff also need to realize that personalized care involves the whole person, from psychological to specific physical needs, after their stroke. Whole-person nursing for stroke patients with physical, mental, social and spiritual problems can help alleviate their negative emotions, improve their coping ability, and promote their physical and mental rehabilitation (49).

## Data Availability Statement

The original contributions presented in the study are included in the article/supplementary material, further inquiries can be directed to the corresponding authors.

## Ethics Statement

The studies involving human participants were reviewed and approved by Ethics Committee of General Hospital of Southern Theatre Command. The patients/participants provided their written informed consent to participate in this study.

## Author Contributions

ML: responsible for the writing and overall design of the manuscript. W-jZ: participated in the content integration. ML: acquisition of data. QL and YD: analysis and interpretation of the data. HC: statistical analysis. H-zX: critical revision of the manuscript for intellectual content. All authors contributed to the article and approved the submitted version.

## Funding

This study was supported by Science and Technology Program of Guangzhou, China (201704020155), Military Health Care Project, China (16BJZ58), and Joint Training Graduate Student Demonstration Base Project [Grant No. Teaching and Research of Guangdong (2018) No. 17].

## Conflict of Interest

The authors declare that the research was conducted in the absence of any commercial or financial relationships that could be construed as a potential conflict of interest.

## Publisher's Note

All claims expressed in this article are solely those of the authors and do not necessarily represent those of their affiliated organizations, or those of the publisher, the editors and the reviewers. Any product that may be evaluated in this article, or claim that may be made by its manufacturer, is not guaranteed or endorsed by the publisher.
